# Screening Tools as a Predictor of Injury in Dance: Systematic Literature Review and Meta-analysis

**DOI:** 10.1186/s40798-018-0146-z

**Published:** 2018-07-18

**Authors:** Ross Armstrong, Nicola Relph

**Affiliations:** 1 Sports Injuries Research Group, Department of Sport and Physical Activity, Edge Hill University, Ormskirk, Lancashire L39 4QP England; 2 Faculty of Health and Social Care, Edge Hill University, Ormskirk, Lancashire L39 4QP England

**Keywords:** Screening tool, Dancers, Musculoskeletal, Injury prevention, Injury risk, Compensated turnout, Functional turnout, Hip range of motion

## Abstract

**Background:**

Dance involves movements of complexity and physical intensity which result in stress on the body. As a consequence, dancers are at risk of injury which can impact on their well-being. Screening tools are used for injury prevention to identify those dancers at risk of injury. The aim of this study was to investigate which screening tools can predict injury in dancers, encompassing all dance genres, levels and ages.

**Methods:**

An electronic search of seven databases from their inception to December 2017 was conducted. The databases were the Allied and Complementary Medicine Database (AMED), CINAHL, eBOOK Collection (EBSCOhost), MEDLINE, Cochrane Database of Systematic Reviews, SPORTDiscus and PEDro: the Physiotherapy Evidence Base. The following search terms were used: (i) Dance AND injury AND Screening, (ii) Screening AND dance and (iii) Musculoskeletal AND Screening AND Dance. Studies were assessed using a 20-point scoring tool, and eligible studies were included in a meta-analysis.

**Results:**

The mean methodological quality score was 12.2 points. Injured dancers had a significantly higher compensated turnout range of motion than non-injured dancers: pooled mean difference of compensated turnout (23.29°; 95% CI 14.85–31.73; *P* < 0.00001; *I*^2^ = 0%). Injured dancers had significantly greater functional turnout range of motion when compared to non-injured dancers: pooled mean difference of functional turnout (14.08°; 95% CI 7.09–21.07; *P* < 0.0001; *I*^2^ = 0%). There also some evidence for use of hip range of motion as a predictor of dance injury.

**Conclusions:**

Some evidence exists for the potential use of dance-specific positions as a predictor of injury. A number of studies were limited by a lack of prospective injury design, injury definition and self-reporting of injury.

## Key Points

- Functional turnout and compensated turnout range of motion may predict injury in dancers.
- Some evidence exists for measurement of hip range of motion as a predictor of injury.
- There is a need for prospective studies that define the injury and have a physical therapist/physiotherapist and/or doctor providing the diagnosis of the injury.

## Background

Dance is an intermittent exercise associated with short sets of explosive movements that require balance, athleticism and artistry [1], indicative of the movement complexity and intensity. Therefore, dancers require physical attributes including strength, speed, power, agility, cardiovascular endurance, flexibility, coordination and balance to meet the performance demands. Dance places considerable stress on the body; vertical ground reaction forces increase with the intensity of the dance routine [2, 3], and mechanical loading increases with movement difficulty [4]. These high forces generated during dance combined with movements that often exceed normal anatomical range can potentially result in injury [5].

Dance injury rates between 0.62 and 5.6 injuries per 1000-h dance have been reported [5–8]. The majority of injuries occur in the lower limb with overuse and foot and ankle injuries most prevalent [5–8]. The demands of dance are varied, for example, ballet requires partner lifting, tap dancing uses the lower extremity as a percussion tool and upper limb weight bearing is required in breaking. Previous systematic reviews [9, 10] have highlighted that dance has a high risk of injury regardless of genre and level. One possible cause is repetitive poor movement patterns which may result in micro-trauma and subsequent injury [11]. The negative impact that injury can have on a dancer’s health and well-being means that injury prevention practices are crucial. These injury prevention practices require collaboration within the Sports Medicine Team which may include physiotherapists/physical therapists, doctors, rheumatologists, nurse practitioners, strength and conditioning coaches and sports scientists.

Screening tools are a vital component of injury prevention that may identify athletes that are at risk of injury development [12–16]. Tools include scales that grade movements such as the Functional Movement Screen [17, 18], the Star Excursion Balance Test [19] and the Beighton Score [20] or the recording of specific joint measurements such as range of motion (ROM). The development of screening tools often utilise the Van Mechelen model of injury prevention [21] and injury audit which can assist in the development of injury prevention programmes.

The determination and implementation of effective screening tools could have positive physical and psychological impact on dancers by allowing participation with reduced injury risk. There is an abundance of literature considering screening tools in dance; however, the findings of this literature have yet to be synthesised in a systematic review and meta-analysis. The current systematic literature review is the first to investigate which screening tools can predict injury in dancers and encompasses all dance genres, levels and ages. A meta-analysis is also completed to synthesise similar data sets where appropriate.

## Methods

### Literature Search

A systematic literature search was conducted to obtain articles concerning screening tools that can potentially predict injury in dancers from their inception of seven databases until December 2017. The databases were the Allied and Complementary Medicine Database (AMED), CINAHL, eBOOK Collection (EBSCOhost), MEDLINE, Cochrane Database of Systematic Reviews, SPORTDiscus and PEDro: the Physiotherapy Evidence Base. A combination of the following search terms was used: (i) Dance AND injury AND Screening, (ii) Screening AND dance and (iii) Musculoskeletal AND Screening AND Dance. These terms were searched in all text, abstract, title and subject terms. Reference lists of acquired articles were screened to find additional articles, and duplicates were removed. Only peer-reviewed articles in the English language were considered.

### Study Selection

The titles and abstracts of the search returned articles were reviewed by the first author (RA) to identify potential relevance using a two-stage process. The first stage involved the classification of articles as relevant, potentially relevant or irrelevant. During this stage, irrelevant articles were excluded, and articles that met the inclusion were retained for further analysis. The second stage involved the review of the full text of relevant and potentially relevant articles by two reviewers (RA and NR). Both reviewers formulated comments regarding the suitability of articles using the checklist of five inclusion criteria and then met to determine final inclusion via reviewing these comments. Any potential disagreements regarding the inclusion were referred to a third reviewer to determine final inclusion. Studies were included if they were (i) full text, (ii) in the English language, (iii) used a screening tool, (iv) the population was dancers and (v) injury occurrence was reported either retrospectively or prospectively. Studies that utilised equipment such as isokinetic dynamometers, bone mineral density scanners and foot scanners were excluded as they were deemed to be laboratory-based and limited in the practical application of dance injury screening. Studies that used screening to provide a treatment intervention to dancers were excluded.

### Data Extraction

Two reviewers (RA and NR) independently extracted data from each article. The following information was extracted if available: study design (prospective or retrospective), level of evidence, location of testing, inclusion and exclusion criteria, subject characteristics (age, sex, height, weight); screening tool and/or physical measurements recorded; reliability and validity of screening tool and/or physical measurements and method of injury collection including retrospective/prospective injury collection, definition of injury, individual diagnosing injury, statistical analysis of injury measure, percentage of missing data or withdrawals, outcome measures and identification of confounders.

### Methodological Quality

A previous review of injury screening tools in team sports [22] utilised a 16-point scoring system. This scoring tool was developed from a modified version of the Cochrane Group on Screening and Diagnostic Test Methodology (Cochrane methods) [23]. However, limitations in this tool were identified such as reliability analysis; studies that reported reliability using data collected within the study were scored the same as studies which provided reliability values from previous studies. The previous scoring system also failed to acknowledge the importance of providing an injury definition and accurate diagnosis, as highlighted in previous dance injury reviews [9, 10]. The strength of prospective injury study design in comparison with retrospective design and the need for multivariable analysis to identify injury risk factors has been advocated [9]. Therefore, the authors decided to add four points to the scoring tool including (i) definition of injury provided (1 point), (ii) diagnosis of injury by physical therapist/physiotherapist or doctor (1 point), (iii) the use of regression models or risk measurement (1 point) and (iv) reliability reported for the actual study (1 point).

The maximum score of the modified tool was 20 points. The scoring system is outlined in Table 1. The level of evidence devised from the Oxford Centre for Evidence-Based Medicine ranged from 1 to 5, with 1 the lowest and 5 the highest score. With regard to the design of the study, those studies that included both retrospective and prospective injury data collection were awarded 1 point, setting information needed to include the name of the venue and for inclusion and exclusion criteria both had to be stated to score 1 point. The methodological score based on statistical analysis was divided into two separate questions. The study was awarded 1 point if it had included an inferential statistical analysis of any kind. However, the study was awarded an additional point if a regression model or risk measurement had been applied; in the current review, this included linear regression models, logistical regression models, Cox regression models, odds ratio (OR) analysis and relative risk (RR) analysis. This aspect of the methodological quality score would allow differentiation between the studies that consider the injury screening tool predictive capability and those who did not. The studies which considered only the ability of the screening tool to identify the differences between the injured and non-injured groups were not awarded with this additional point.

**Table 1:** Methodological quality score for each study

Study	Design^a^ (1)	Level of evidence^b^ (5)	Selection criteria^c^ (1)	Setting^d^ (1)	Demographic information^e^ (1)	Description of screening tool^f^ (2)	Injury definition^g^ (1)	Injury diagnosis^h^ (1)	Statistical analysis^i^ (1)	Predictive statistical analysis^j^ (1)	Reliability of index test^k^ (2)	Percentage missing^l^ (1)	Outcome^m^ (1)	Confounders^n^ (1)	Total score (20)
Luke et al. [5]	1	4	0	1	1	2	0	0	1	0	0	0	1	0	11
Coplan [24]	0	4	0	1	1	2	1	0	1	0	0	0	1	0	11
Hamilton et al. [26]	1	4	0	1	1	1	0	1	0	0	0	0	1	0	10
Allen et al. [27]	1	4	0	1	1	2	1	1	1	0	0	0	1	0	13
Gamboa et al. [28]	0	4	0	1	1	2	1	1	1	1	1	0	1	0	14
Hamilton et al. [29]	0	4	1	1	1	2	0	0	0	0	0	0	1	0	10
Negus et al. [30]	0	4	1	1	1	2	1	0	1	0	2	0	1	0	14
Zaletel et al. [31]	0	4	0	1	0	1	1	0	1	1	2	0	1	0	12
Bhakay et al. [32]	0	4	1	1	0	2	1	0	1	0	0	0	1	0	11
Wong et al. [33]	0	4	0	1	0	0	0	0	0	0	0	0	1	0	6
Thomas et al. [34]	0	4	0	1	1	0	0	0	1	0	0	1	1	0	9
Drężewska et al. [35]	1	4	1	1	1	1	0	0	1	0	0	0	1	0	11
Twitchett et al. [36]	0	4	1	0	1	2	0	0	1	0	0	0	1	0	10
McCormack et al. [37]	0	4	0	1	0	1	0	0	1	1	0	0	1	0	9
Bowerman et al. [38]	1	4	0	1	1	2	1	1	1	1	0	1	1	0	15
Lin et al. [39]	0	4	1	1	1	2	1	0	1	0	0	0	1	0	12
Frusztajer et al. [40]	0	4	0	0	1	0	0	0	1	0	0	0	1	0	7
Watkins et al. [41]	0	4	1	1	1	2	0	0	1	1	1	0	1	0	13
McNeal et al. [42]	0	4	1	1	1	2	0	0	1	0	1	0	1	0	12
Reid et al. [43]	0	4	0	1	1	2	0	0	1	0	2	0	1	0	12
Baker-Jenkins et al. [44]	1	4	1	1	1	1	1	1	1	1	0	0	1	0	14
Ruemper and Watkins [45]	0	4	0	1	1	2	1	0	1	0	0	0	1	0	11
Cahalan et al. [46]	0	4	1	1	1	2	1	0	1	1	0	0	1	0	13
Cahalan et al. [47]	1	4	1	1	1	2	1	0	1	1	0	1	1	0	15
Cahalan et al. [48]	1	4	1	1	1	2	1	0	1	0	0	0	1	0	13
Steinberg et al. [49]	0	4	0	1	1	2	0	1	1	1	1	0	1	0	13
Jacobs et al. [50]	0	4	1	1	1	1	1	0	1	1	0	0	1	0	12
Martin et al. [51]	0	4	0	1	0	0	1	0	0	0	0	0	0	0	6
Angioi et al. [52]	1	4	0	0	1	2	1	0	1	1	0	0	1	0	12
Hiller et al. [53]	1	4	1	1	1	2	1	0	1	1	1	1	1	0	16
Van Merkensteijn et al. [54]	0	4	0	1	1	2	1	0	1	0	0	0	1	0	11
Wiesler et al. [55]	0	4	0	1	1	2	1	1	1	1	0	0	1	1	14
Kenny et al. [56]	0	4	1	1	1	2	1	0	1	1	0	1	1	0	14
Lee et al. [57]	1	4	0	1	1	2	1	0	1	1	2	1	1	0	16
Davenport et al. [58]	1	4	1	1	1	1	1	0	1	0	0	1	1	0	13
Roussel et al. [59]	0	4	0	1	1	2	1	0	1	0	0	1	1	0	12
Twitchett et al. [60]	1	4	0	0	1	2	0	1	1	1	0	0	1	1	13
Steinberg et al. [61]	0	4	1	1	1	2	0	0	1	1	0	0	1	0	12
Roussel et al. [62]	1	4	0	1	1	2	1	0	1	1	2	0	1	0	15
Steinberg et al. [63]	0	4	1	1	1	2	1	1	1	1	2	0	1	1	17
Steinberg et al. [64]	1	4	0	0	1	2	1	1	1	1	2	0	2	0	16
Van Seters et al. [65]	1	4	1	1	1	2	1	0	1	1	1	1	1	0	16

The maximum possible score for quality was 20; this score was derived from 14 domains

^a^Study design (1 point = prospective, 0 point = retrospective)

^b^Level of evidence (Oxford Centre for Evidence-Based Medicine Levels of Evidence: level 1 = 5 points; level 2 = 4 points; level 3 = 3 points; level 4 = 2 points; level 5 = 1 point)

^c^Selection criteria (inclusion and exclusion criteria were clearly described = 1 point)

^d^Setting (enough information was provided to identify the setting = 1 point)

^e^Demographic information (age (mean or median and SD or range) and gender were reported = 1 point)

^f^Description of the screening tool (test device or instruments = 1 point, protocol of screening tool(s) reported = 1 point, insufficient data to permit replication of the test)

^g^Injury definition (clear and appropriate definition is provided = 1 point)

^h^Injury diagnosis (made by physical therapist/physiotherapist or doctor = 1 point, self-assessed = 0 point)

^i^Statistical analysis (detail given on mean or median, SD, *P* value or CI = 1 point)

^j^Predictive statistical analysis (multivariate regression analysis or RR/OR used as predictive value = 1 point)

^k^Reliability of index test (reliability reported from previous research = 1 point, reliability reported from actual study data = 2 points)

^l^Percentage missing (all included subjects measured and if appropriate missing data or withdrawals from a study reported or explained = 1 point)

^m^Outcome (outcome clearly defined and method of examination of outcome adequate = 1 point)

^n^Confounder (most important confounders and prognostic factors identified and adequately taken into account in design study = 1 point)

### Data Extraction and Analysis

Studies that included similar screening tools were considered for inclusion in the meta-analysis. The following data were extracted by one reviewer and cross-checked by the second reviewer: the number of participants, mean screening tool measurement and accompanying standard deviations. It was possible to synthesise the data from three screening tools reported in the included studies, all of which related to turnout. In ballet, turnout refers to the outward rotation of the legs and feet so that the hips are externally rotated and is required to achieve first, second, third, fourth and fifth ballet positions [24]. The following comparisons were possible in the current review:

- Passive hip external rotation range of motion in the injured group vs passive hip external rotation range of motion in the non-injured group.
- Functional turnout (defined as the angle of turnout assumed by a dancer in any of the five basic ballet positions [24]) in the injured group vs function turnout in the non-injured group.
- Compensated turnout (defined as the difference between the first position turnout angle (functional turnout) and the total ROM of passive hip external rotation for both hips [24]) in injured group vs compensated turnout in the non-injured group.

Comparisons were made using a fixed effect model with an inverse variance method and presented as forest plots using Review Manager Software (version 5.3.5). The mean difference between groups measured the effect size. Heterogeneity between comparable trials was tested using the chi-squared test (level of significance set at *P* < 0.10) and *I*^2^ percentages (lower than 50%) [25]. Studies that could not be included in the meta-analysis were analysed using qualitative review.

## Results

### Included Studies

**Fig. 1:**
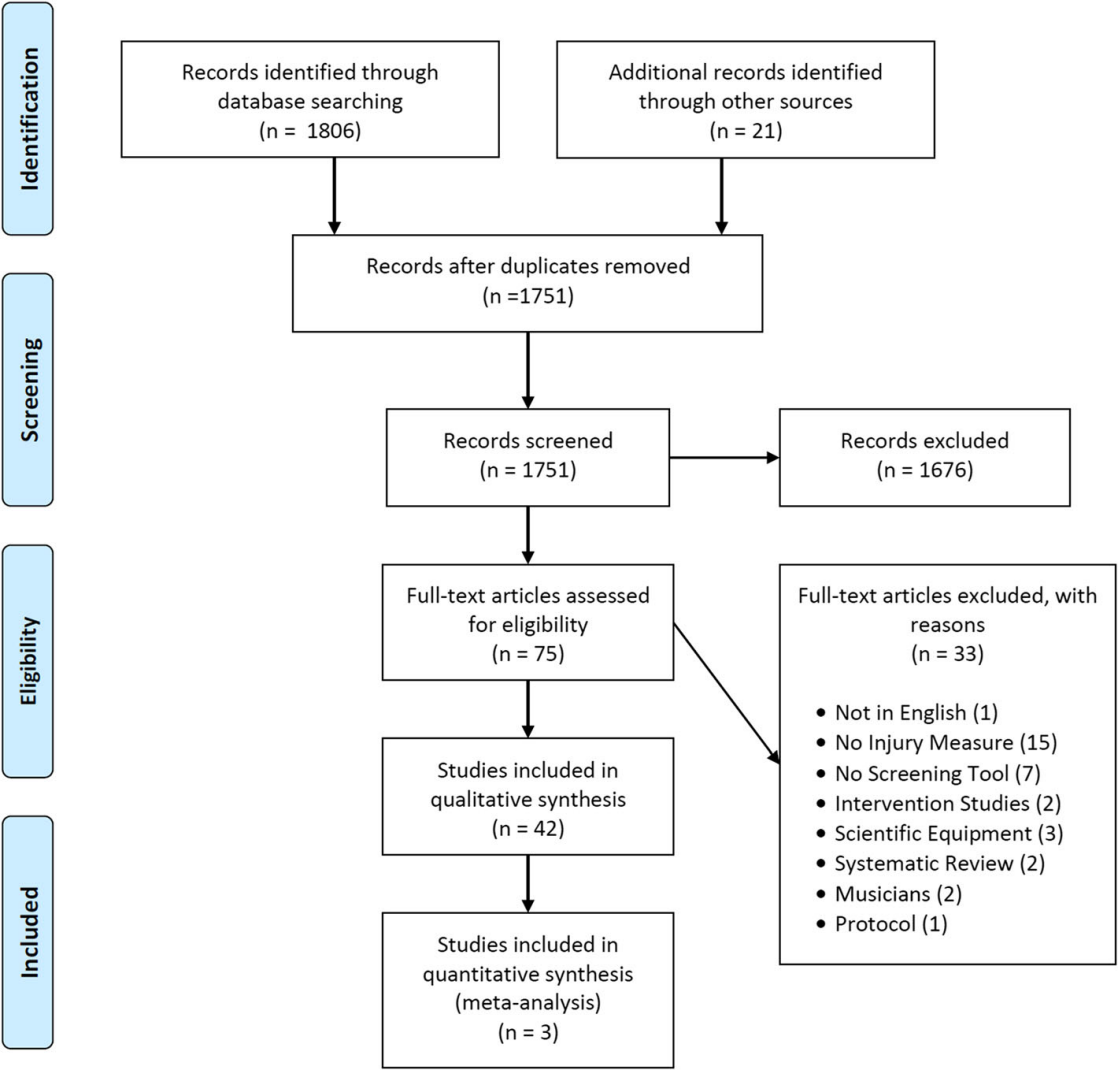
A PRISMA diagram of the search strategy

The initial search yielded 1806 studies for review. The title and abstracts of these articles were reviewed and duplicates removed, which resulted in 75 articles requiring further consideration. Assessment of the eligibility of the full text of these articles and the application of inclusion and exclusion criteria meant that 42 articles were included in the systematic review. Figure 1 outlines the search strategy. The assessment of the methodological quality is reported in Table 1. The mean score was 12.2 points (range 6–17 points). Table 2 reports the characteristics of these studies.

**Table 2:** Characteristics of the studies included in the literature review

Article	Population	Screening tools	Definition of injury	Diagnosis of injury	Findings
Luke et al. [5]	*N* = 39 (34 females) Male and female pre-professional dancers Aged 14–18 years	• Marshall Test/Micheli Score • Scoliosis • Ankle plantarflexion (PF) and dorsiflexion (DF) • Foot arch and type • First metatarsophalangeal angle • Hip internal and external rotation • Popliteal angle • Hip flexor (Thomas test) • Iliotibial band (Ober’s) • Q angle • Leg length • Foot/thigh angle	No definition of injury provided Categorised as: New, recurrent, overuse and soft tissue	Self-reported	No correlations to injuries except age, sex and popliteal angle. Left popliteal angle was related to self-reported injury (*r* = 0.340, *P* = 0.03)
Coplan [24]	*N* = 30 (27 females) College students and teachers Injured males 20 ± 0 years 183 ± 0 cm 69 ± 0 kg Non-injured males 27 ± 7 years 190 ± 14.1 cm 83 ± 11.2 kg Injured females 19.7 ± 1.6 years 163.2 ± 4.1 cm 53.2 ± 5 kg Non-injured females 23.8 ± 8.7 years 162 ± 4 cm 54.9 ± 6.2 kg	• Passive hip internal and external rotation • 1st position turnout • Functional turnout (the angle of turnout assumed by a dancer in any of the five basic ballet positions) • Compensated turnout (difference between measured functional turnout and total passive external rotation)	‘Any pain or dysfunction of the low back or lower extremities that impacted ability to perform’	Self-reported by questionnaire	Significant difference between injured and non-injured groups for functional turnout (*P* = 0.004) and compensated turnout (*P* = 0.006)
Hamilton et al. [26]	*N* = 40 females Elite dancers at the School of American Ballet 14.92 ± 0.96 years 93% Caucasian 5% Asian 2% Hispanic	• Leg length • Scoliosis • Axial alignment • First position • Second position • Fifth position • Sauté • Spondylolisthesis (lumbosacral step off) • Hip motion (prone) • Turnout • Quadriceps tightness • Hamstring tightness • Recurvatum • Patellar alignment • Patella tendinitis • Ankle PF • PF sign • Peroneal weakness • Foot type • Turning preference	Categorised according to nature and duration of disability and frequency of occurrence but no specific definition provided	Orthopaedist recorded injury history	Minor injured dancers had lack of turnout noted in asymmetry in grande plié (12 v 0%, *P* < 0.05), unequal hip motion (37 v 16%, *P* < 0.05) and pronation when landing from sauté (62 v 25%, *P* < 0.05)
Allen et al. [27]	*N* = 52 year 1 *N* = 58 year 2 *N* = 53 year 3 Elite, professional ballet company Year 1 Male 23 ± 4 years 179 ± 4.3 cm 71.5 ± 4.7 kg Female 25 ± 5 years 162 ± 3.9 cm 49.2 ± 4.04 kg Year 2 Male 24 ± 4 years 179 ± 1.0 cm 71.5 ± 4.73 kg Female 25 ± 5 years 162 ± 0.96 cm 49.2 ± 4.05 kg Year 3 Male 24 ± 4 years 179 ± 5.3 cm 72.7 ± 7.01 kg Female 26 ± 5 years 164 ± 3.6 cm 51.2 ± 5.59 kg	• Years of training • Functional Movement Screen	‘Any injury that prevented a dancer from taking full part in all dance-related activities for a period of greater than or equal to 24 h after the injury was sustained’ Categorised as: Traumatic Overuse Recurrent	Physiotherapist diagnosed injury	Injury incidence declined from year 1 (4.76/1000 h) to year 2 (2.40/1000 h) and year 3 (1.81/1000 h) (*P* < 0.001)
Gamboa et al. [28]	*N* = 151 females Elite pre-professional boarding ballet school dancers Injured (*n* = 72) 14.3 ± 1.8 years Non-injured (*n* = 125) 14.4 ± 2.1 years	• Posture (forward head, cervical lordosis, thoracic kyphosis, lumbar lordosis, scoliosis, knee hyperextension, foot position) • Strength (upper, lower, core trunk, core scapula) • Flexibility (upper and lower) • Orthopaedic testing (knee, foot, hip, ankle) • Function (turnout × 3, gesture leg turnout, releve balance time, developpe test, plié turnout alignment, heel raise, pelvic alignment, releve´ raise during developpe)	‘When a dancer sought at least 1 treatment session from the physical therapist’	Physical therapist diagnosed injury	Significant differences between injured and non-injured groups for right foot pronation (*P* = 0.005), lower extremity strength (*P* = 0.045) and ankle PF on the right side (*P* = 0.037)
Hamilton et al. [29]	*N* = 28 (14 females) Elite American Ballet Theatre Female 29.23 ± 5.25 years Male 28.42 ± 4.08 years	• Flexibility (elbow hyperextension, external arm rotation, lotus, external leg rotation, knee recurvatum, palms to floor) • Strength (hip abductors, adductors, abduction/adduction ratio, knee extensors, flexors, ankle PF, DF, PF:DF ratio) • Range of motion (ROM) (hip external/internal rotation, hip abduction/ adduction, hip flexion, knee hyperextension, tibial torsion, tibial external rotation, tibial internal rotation, ankle plantarflexion/dorsiflexion)	No definition of injury provided	Self-reported by questionnaire	Males with 4 or more past injuries were more flexible (increased elbow extension *P* < 0.003 and straight leg raise *P* < 0.05). Overuse history increased ability to perform the lotus *P* < 0.005 and increased total turnout *P* < 0.005 Females with more injuries had less turnout (*P* < 0.005). Overuse injuries were related to less bilateral plié (*P* < 0.001) and decreased left ankle DF (P < 0.05)
Negus et al. [30]	*N* = 29 (24 females) Student dancers Aged 15–22 years	• Hip external rotation ROM in supine (passive and active) • Functional turnout in standing • Active external rotation lag • Compensated turnout (CT) (= static functional turnout angle (standing in 1st, 5th right,5th left)—total active hip external rotation (supine)) • Static-dynamic turnout (SDTD) (= standing in 1st,5th right,5th left)—dynamic functional turnout angle (landing in same 3 positions after jumping)	‘Any pain, discomfort or other musculoskeletal problem, which required modification of, or time away from, dance training, examinations, or performance’ Categorised as traumatic and non-traumatic	Self-reported VAS scale used to self-report severity and perceived impact of injury	Number of non-traumatic injuries was positively correlated with 6 of 7 derived turnout variables; compensated turnout in all 3 positions and static dynamic turnout difference in all 3 positions (*r* = 0.39–0.55, *P* < 0.039). Severity of non-traumatic injuries was positively correlated with 3 of 7 derived turnout variables: static-dynamic turnout difference in all positions (rho = 0.38–0.47, *P* < 0.043) Non-traumatic injuries— CT first, right fifth, left fifth, SDTD first, SDTD right fifth, SDTD left fifth correlated with number of injuries SDTD first, SDTD right fifth, SDTD left fifth correlated with injury severity No correlations to traumatic injury
Zaletel et al. [31]	*N* = 24 females Ballet high school students 16–18 years 165.3 ± 5.7 cm 55.2 ± 5.4 kg	• Body mass • Height • Skinfolds: triceps, subscapular, calf, suprailiac	‘Any physical complaint sustained as a result of performance or training, irrespective of the need for medical attention or time lost from activity’	Self-reported by questionnaire	Increased likelihood of ankle injuries for endomorphs (OR = 1.887) Increased likelihood of foot injury for ectomorph (OR = 1.719) Toe injuries more prevalent in dancers with higher body mass (OR = 1.688)
Bhakay et al. [32]	*N* = 22 females Professional ballet dancers attending Indian dance schools 14–30 years	• External hip rotation • Functional turnout • Compensated turnout	‘Any pain or dysfunction of the lower extremities that impacted the dancers’ ability to practice or perform’	Self-reported	Relationship between total hip external rotation (*P* = 0.0137), functional turnout (*P* = 0.0176) and compensated turnout (*P* = 0.0002) and injury
Wong et al. [33]	*N* = 207 Student dancers resident at a pre-professional ballet academy	• Muscle strength • Flexibility • Alingnment • Posture • History of injury • Ankle foot risk screening scores	No definition of injury provided	Not reported	A screening score of ≥ 19 was attributed to being ‘at risk’ of injury
Thomas et al. [34]	*N* = 239 females Ballet students attending intensive summer ballet programmes 15 ± 1.5 years	• Body mass index (BMI)	No definition of injury provided	Self-reported	No difference in BMI between injured and non-injured groups
Drężewska et al. [35]	*N* = 71 (45 females) Enrolled ballet school students 16.5 years	• Sacrum inclination angle • BMI	No definition of injury provided	Self-reported	A comparison of sacral inclination angles in a position with the feet placed parallel and in the turnout position showed statistically significant changes in the angle among respondents reporting pain (*P* < 0.05) Dancer with sacrum inclination angles of ≥ 30° had higher mean pain intensity scores Pain was more frequent in female dancers whose BMI was lower than normal (< 18.5 kg m^2^)
Twitchett et al. [36]	*N* = 42 (31 females) Ballet students in vocational training 17.3 ± 1.02 years	• Somatotype • Skin fold thickness • Body composition	No definition of injury provided	Self-reported by questionnaire	Ectomorphy was a strong predictor of the number of acute injuries sustained (*P* = 0.026); these parameters had a significant negative correlation (*r* = − 0.37, *P* = 0.016) Correlations were observed between the dancers’ total time off due to injury and %body fat (*r* = − 0.31, *P* = 0.048) and between the total time off resulting from acute injury and both %body fat (*r* = − 0.32, *P* = 0.04) and ectomorphy *r* = − 0.42, *P* = 0.005) The number of overuse injuries sustained and time off due to overuse injury were also correlated with mesomorphy (*r* = − 0.38, *P* = 0.015 and *r* = − 0.33, *P* = 0.032, respectively)
McCormack et al. [37]	*N* = 70 (38 females) Dance students at the Royal Ballet School, London	• Height • Weight • Lower segment length • Arm span • Beighton and Contompasis scores	No definition of injury provided	Unclear	18 female and 12 male dancers exhibited features (as well as hypermobility and joint pain) to satisfy Brighton Criteria (OR 6.75 CI 1.35–33.66) and (OR 7.8 CI 0.90–67.37)
Bowerman et al. [38]	*N* = 46 (30 females) Australian ballet School students 16 ± 1.58 years	• Maturation tanner scale • Height • Body mass • Foot length for growth • Lower extremity alignment during fondu and temps leve	‘Any physical harm resulting in pain or discomfort that required a dancer to modify their dance activity during one or more classes, or which required a dancer to cease all dance related activity’ Only injuries that occurred as a result of dance training were located in the lumbar spine and/or lower extremities of the body and were an overuse injury were included	Physiotherapist diagnosed injury	Changes in right foot length (RR = 1.41, CI = 0.93–2.13), right knee angles during the fondu (RR = 0.68, CI = 0.45–1.03) and temps levé (RR = 0.72, CI = 0.53–0.98), and pelvic angles during the temps levé on the left (RR = 0.52, CI = 0.30–0.90) and fondu on the right (RR = 1.28, CI = 0.91–1.80) were associated with substantial changes in injury risk
Lin et al. [39]	*N* = 22 females Ballet school students Injured dancers 19.7 ± 2.4 years Uninjured dancers 18.8 ± 3.1 years	• Height • Weight • Active and passive ROM (ankle, knee and hip joints) • Standing turnout angle • Anterior draw • Talar tilt	‘…1 or more ankle sprains related to ballet dancing within the past year that interrupted dance training or rehearsal for at least 24 h’	Self-reported	No significant difference in any of the physical measures
Frusztajer et al. [40]	*N* = 50 female dancers Classical ballet dancers 20.5 ± 3.9 years (range 16–29 years)	• Height • Weight	No definition of injury provided	Subjects interviewed by a nurse practitioner on present and past illness and fractures	The mean weight of the group with stress fractures fluctuated to a significantly lower weight, 80% of dancers reaching a low weight, at least 25% below ideal (*P* < 0.005)
Watkins et al. [41]	*N* = 350 (286 females) Dance students from three pre-professional ballet schools in New England Age ranged from 11.1 to 23.2 years	• Turnout alingnment • Angle of deviation	No definition of injury provided	Self-reported by questionnaire	No significant relationship between deviation in alingnment and injury rate for knee, ankle or foot
McNeal et al. [42]	*N* = 350 (286 females) Dance students from three pre-professional ballet schools in New England Age ranged from 11.1 to 23.2 years	• Turnout alingnment • Angle of deviation	No definition of injury provided	Self-reported by questionnaire	No significant relationship between deviation in alingnment and injury rate for knee, ankle or foot
Reid et al. [43]	*N* = 30 females Dancers from a local ballet school 15.4 years (range 13–19 years)	• ROM (passive hip flexion, extension, adduction, abduction, internal rotation and external rotation, knee extension)	No definition of injury provided	Interviews used to diagnose injury	Passive hip abduction was significantly reduced in dancers with lateral pain or snapping hip (*P* = 0.05)
Baker-Jenkins et al. [44]	*N* = 47 Female Student dancers 19.9 ± 2.51 years 165 ± 0.05 cm 56.23 ± 6.51 kg	• Functional turnout • Total passive turnout • Passive hip external rotation • Compensated turnout • Active external rotation • Muscular = functional turnout/passive hip external rotation	‘Physical damage to the body or body part which prevented completion of one or more entire curriculum class’	Physiotherapist diagnosed injury	Compensated turnout and muscular predictors of being in the 2+ injury group For every 1% increase in compensated turnout, 9% increase in the odds of being in the 2+ group 1% increase in muscular = 8.4% increase in the odds of being in the 2+ group For every 1% increase in compensated and muscular values, there was a corresponding 9% increase in odds that the dancer would sustain 2 or more injuries compared to 0 or 1 injury
Ruemper and Watkins [45]	*N* = 85 (78 females) Contemporary dance students Year 1: Males 19.33 ± 0.57 years Females 20.14 ± 1.96 years Year 3: Males 23.00 ± 2.16 years Females 22.59 ± 2.15 years	• Beighton Score • Brighton Criteria • Height • Weight	Physical complaint injury: (1) ability to perform full dance activities; (2) Attended a triage session but not a physiotherapy session Medical injury: ‘an injury resulting in medical attention (physio, etc.) beyond triage’ Time loss injury: ‘an injury resulting in inability to participate in activities (class etc.)’	Self-reported by questionnaire	The total number of injuries and time loss injuries were correlated with Brighton Criteria (*P* = 0.001) Physical complaint injuries and Brighton Criteria were correlated (*P* = 0.005) Time loss injuries were related to joint hypermobility syndrome (*P* = 0.001)
Cahalan et al. [46]	*N* = 104 injury questionnaire *N* = 84 physical assessment Elite, competitive and student Irish dancers Professional (*n* = 36) 23 years 50% female Student (*n* = 28) 20 years 85.7% female Competitive (*n* = 40) 20 years 80% female	• BMI • Waist:hip ratio • Pain pressure threshold • Navicular drop • Functional Movement Screen (total of deep squat and in-line lunge scores only) • Hamstring flexibility • Gastrocnemius flexibility • Star Excursion Balance Test (SEBT) Posteromedial reach • Vertical leap • Beighton Score • Number of jumps in 30s • (% max HR) • Type and frequency of cross-training	Time loss definition of injury categorised as: Minor injuries (up to 7 days to resolve) Moderate injuries (8 to 21 days to resolve) Severe (21 days + days to resolve)	Self-reported by questionnaire	No significant differences between injured and non-injured groups
Cahalan et al. [47]	*N* = 85 (66 female) Elite, competitive and student Irish dancers Divided in to more time absent (MTA) from injury (*n* = 41, 20 years) and less time absent (LTA) from injury (*n* = 25, 20 years)	• BMI • Waist:hip ratio • Navicular drop • Functional Movement Screen total score • Hamstring flexibility • Gastrocnemius flexibility • SEBT Posteromedial reach • Vertical leap • Beighton Score • Number of jumps per 30 s • % max heart rate • Type and frequency of cross-training	‘Any physical complaint that caused absence from one or more rehearsals or performance days’	Self-reported by questionnaire	‘More time absent’ (MTA) group demonstrated a trend towards better performance on Functional Movement Screen (*P* = 0.062)
Cahalan et al. [48]	*N* = 85 (66 females) Elite, competitive and student Irish dancers Divided in to MTA from injury (*n* = 41, 20 years) and ‘less time absent’ from injury (*n* = 25, 20 years)	• BMI • Waist:hip ratio • Navicular drop • Functional Movement Screen total score • Hamstring flexibility • Gastrocnemius flexibility • Balance • Vertical leap • Beighton score • Number of jumps per 30 s	‘Any physical complaint that caused absence from one or more rehearsal or performance days’	Self-reported by questionnaire	No significant differences between the injured and non-injured groups
Steinberg et al. [49]	*N* = 1288 Females Non-professional Aged 8–16 years	• ROM (ankle and foot en-pointe, ankle PF, hip external rotation and abduction, lower back flexibility, hamstring flexibility). • Anatomical: knee valgus, knee varum, splay foot, forefoot adduction, hindfoot varum, hindfoot valgus, longitudinal arch planus, scoliosis, lordosis • Dance technique: releve, turnout, plié • Height and weight	No definition of injury provided	Orthopaedic surgeon specialising in dance medicine diagnosed injury	Dancers with foot or ankle tendonopathies and dancers with non-categorised injuries manifested hyper hip abduction ROM (*P* = 0.002) Scoliosis was significantly related to injury for 8 to 12 years (*P* < 0.01) and 13 to 16 years (*P* < 0.01) age groups Amount of en-pointe (> 60 min/week) related to injury (*P* < 0.001) For knee injuries, ankle PF (*P* = 0.002) and hip abduction (*P* = 0.0033) were significant predictors of injury For hip abduction, ROM (*P* < 0.001) was a significant predictor of injury For back injuries, scoliosis (*P* < 0.001), ankle PF (P = 0.026) and poor dance technique (rolling in) (*P* = 0.021) were significant predictors of injury For non-categorised injuries, ankle PF (*P* = 0.017), hip abduction (P < 0.001) and poor dance technique (rolling in) (*P* = 0.002) were significant predictors of injury
Jacobs et al. [50]	*N* = 260 (145 females) Professional ballet dancers in 4 countries 178 ballet, 82 modern Median age range 21–30 years	• BMI	‘…functional inability due to pain’	Self-reported by questionnaire	No significant findings for BMI reported
Martin et al. [51]	*N* = 158 Dancers	• Ankle ROM	‘Those severe enough to require medical attention and cause at least 1 day of missed rehearsal’	Self-reported	Dancers with previous injuries had significantly lower flexibility. Ankle flexibility was not an injury predictor
Angioi et al. [52]	*N* = 16 females Professional contemporary dancers dance students 26 ± 4.7 years 165.3 ± 4.8 cm 59.2 ± 7.6 kg	• Anthropometry • Flexibility (developpe a la seconde, combined hip flexion, and abduction and external rotation) • Muscle power • Muscle endurance • DAFT	If ‘…they were unable to take part in class, rehearsals or performance in the previous 12 months’	Self-reported by questionnaire	There was a significant negative correlation between mean score total days off and standing vertical jump (*r* = − 0.66, *P* = 0.014) The strongest predictor of total days off was standing vertical jump (muscle power) (*P* = 0.014)
Hiller et al. [53]	*N* = 115 (94 females) Student dancers at a performing arts secondary school and local dance school 4.2 ± 1.8 years	• Ankle inversion, eversion, DF and first metatarsophalangeal extension range • Ankle anterior draw laxity (0–3 scale) • Balance (no. of foot lifts in 30 s) • External hip rotation • Cumberland ankle instability tool (0–30 scale) • Balance on demi pointe for 5 s (yes/no)	An ankle sprain: ‘…an inversion injury that had resulted in either swelling or bruising in the area and limping for more than 1 day’	Self-reported	Increased passive inversion range (HR = 1.06) and inability to balance on demipointe (HR = 3.75) increased the risk of injury
Van Merkensteijn et al. [54]	*N* = 15 (9 females) University-level dancers Females: 21.15 ± 1.268 years 167.45 ± 6.168 cm 60.90 ± 5.428 kg Males: 22.50 ± 0.707 years 179.00 ± 12.728 cm 72 ± 4.24 kg	• Active hip external rotation • Functional turnout • Compensated turnout	‘Any pain, discomfort or musculoskeletal problem that would cause modification of technique or time away from dance class, rehearsal or performance. Only dance-related injuries were analysed’	Self-reported by questionnaire	Compensated turnout was related to experiencing more than one injury (*r* = 0.45, *P* = 0.004) There was a relationship between increased compensated turnout and lower back pain (*r* = 0.50, *P* = 0.02)
Wiesler et al. [55]	*N* = 148 (119 female) Dance students at the North Carolina School of Arts *N* = 101 ballet *N* = 47 modern Non-injured dancers 17.41 ± 0.41 years, BMI 19.16 ± 0.32 kg m^2^ Injured dancers 17.75 ± 0.27 years, BMI 19.16 ± 0.25 kg m^2^	• ROM (ankle inversion, eversion, PF, DF, 1st metatarsophalangeal joint PF, DF and hallux valgus)	‘Any acute or chronic problem warranted attention by the aforementioned healthcare professional’	Physical therapist diagnosed injury	Previous injury was predictive of a new injury (*P* = 0.020) Previously injured dancers had significantly lower ankle DF on the corresponding lower limb
Kenny et al. [56]	*N* = 155 (90 females) Pre-professional full-time ballet students at two institutions in Calgary Median age 15 years (range 11–19 years) *N* = 65 (63 females) Pre-professional full-time contemporary students at two institutions in Calgary Median age 20 years (range 17–30)	• Previous training • Previous injury in last 1 year • Irregular menses • %BMI < 18.5 • Low total bone mineral density (% < − 2.0 *z*-score) • Ankle DF ROM • Ankle PF ROM • Active standing turnout • Active straight leg raise (% with impairment) • Knee lift abdominal test (%anterior tilt) • One-leg standing test (% hip hiking) • Unipedal dynamic balance (seconds) • Y Balance Test	‘Any dance related physical complaint that required medical attention and/or time loss (i.e. caused the dancer to miss more than 1 day of class, rehearsal or performance in the previous 1 year)’	Three certified physiotherapists and six kinesiology graduate students administered an injury questionnaire to diagnose injury	Ankle PF ROM in the right ankle was identified as an important covariate
Lee et al. [57]	*N* = 66 (40 females) Dancers 18.15 ± 1.45 years	• Movement competency screen (MCS)	‘Any physical complaint sustained by a dancer resulting from performance, rehearsal or class, and resulting in a dancer injury report or triage irrespective of the need for medical attention or time loss from dance activities’ Categorised into: Time loss, non-time loss, nature of injury, new or recurrent Injury severity: S0: no days off S1: activity modification S2: ≤ 7 days off S3: > 7 days off S4: year ending	Self-reported by questionnaire	MCS score < 23 was an increased risk of injury (*P* = 0.035) Higher number of injuries more likely to be explained by greater number of trunk injuries (*P* = 0.036)
Davenport et al. [58]	*N* = 36 (34 females) Dancers from a University and Arts Conservatory 20.8 ± 1.8 years	• ROM • Hip strength • Core strength • Release swings • Release lunges • Developpe a la seconde	‘Any physical impairment sustained during or because of dance activity that caused the dancer to make different movement choices for the way he/she danced on a given day’	Self-reported by questionnaire	ROM greater than 15% variability between sides was associated with previous injury (*P* = 0.04)
Roussel et al. [59]	*N* = 40 (38 females) Student dancers studying for a professional bachelor degree in Belgium 20.3 ± 2.4 years 1.66 ± 0.06 m 56.43 ± 5.71 kg	• Beighton Score • Lumbopelvic control (knee lift abdominal test, bent knee fall out) • Muscle extensibility • ROM (hip flexion, hip adduction, abduction, Ober’s test) • Pain provocation tests (Patrick’s test, Gaenslen’s test, compression test gapping test)	‘…any trouble’	Self-reported questionnaire	30% of dancers without a history of lower back pain (LBP) were not able to perform a correct contraction of the transversus abdominus muscle compared to 63% of dancers with a history of LBP (*P* = 0.048)
Twitchett et al. [60]	*N* = 13 females Elite dancers who were part of a touring group 19 ± 0.7 years	• Anthropometry • Flexibility • Muscle power • Muscle endurance • Dance Aerobic Fatigue Test (DAFT)	No definition of injury provided	A healthcare professional diagnosed injury	There was a significant positive correlation between number of injuries sustained and heart rate observed at the end of the DAFT (*r* = .590, *P* = 0.034) There was a significant negative association between time modifying their activity due to injury and percentage body fat (*P* = 0.039)
Steinberg et al. [61]	*N* = 806 (588 females) Dancers at centres for advanced training 14.4 ± 2.1 years	• Body structure parameters (standing height, sitting height, low body length, torso length, leg length, arm length, calf girth, thigh girth, upper arm girth, thigh circumference)	No definition of injury provided	Self-reported by questionnaire	Left thigh circumference of injured dancers aged 11–12 years. was significantly larger when compared to non-injured (*P* = 0.005)
Roussel et al. [62]	*N* = 32 (26 females) Student dancers in a full time professional dance programme in Belgium 20 ± 2 years	• Beighton Score • Lumbopelvic movement control (active straight leg raise, bent knee fall out, knee lift abdominal test and standing bow)	‘Any MSK condition requiring time away from dancing’	Self-reported by questionnaire and subjective evaluation	Knee lift abdominal test (*P* = 0.015) and standing bow (*P* = 0.029) were significant predictors of injury
Steinberg et al. [63]	*N* = 271 females Pre-professional dancers at a performing arts centre with patellofemoral pain syndrome (PFPS) Varying ages from 10 to 16 years	• Weight • Height • Leg length • Joint ROM (passive ankle, hip, knee) • Lower back/hamstring flexibility • Knee joint stability • Patella mobility • Anatomical malalignment (knee varum/valgus, hind foot varum/valgus, scoliosis, lordosis)	PFPS was defined as ‘(a) knee pain (at anterior, medial and/or retro patella) during movement or exercises that disturbed their dance practice and daily life activities; (b) the knee pain could be reproduced during physical examination; (c) knee swelling was found; and/or (d) when a positive grinding sign and/or positive patellar inhibition test was obtained when the knee, and especially the patella, was palpated, contracted and stretched’	Unclear	Significantly greater percentage of hindfoot varum (*P* = 0.044) and scoliosis (*P* = 0.15) in the PFPS group Ankle PF was lower and ankle DF and knee flexion ROM were greater in the PFPS group compared with the control group (*P* = 0.005) Factors associated with PFPS among young dancers (aged 10–11 years) were: hip abduction (OR = 0.906), lower back/hamstring flexibility (OR 3.542); among adolescent dancers (12–14 years) were: ankle DF (OR = 0.888), hindfoot varum (OR = 2.66): and in premature dancers (15–16 years) were: ankle PF (OR = 1.060) and hip internal rotation (OR = 1.063)
Steinberg et al. [64]	*N* = 1288 females Non-professional dancers 13.3 years. (range 8–16)	• Weight • Height • Leg length • Foot length • Foot width • BMI • Anatomical anomalies (knee valgus/varus, genu recurvatum, hallux valgus, splay foot, forefoot adduction, hindfoot varum/valgus, longitudinal arch cavus/planus, lordosis)	‘Reproduction of pain and signs of injury (such as swelling)’	Orthopaedic surgeon confirmed injury	The risk of injury was significantly higher for dancers with scoliosis Knee varum (*P* = 0.001), knee hyperextension (*P* = 0.034), long-plantar planus (*P* = 0.038), splay foot (*P* = 0.049) and hallux valgus (*P* = 0.001) values were higher in dancers with scoliosis Back injuries were higher in dancers with scoliosis (*P* < 0.001)
Van Seters et al. [65]	*N* = 28 females First year full-time dance students at Codarts University 8.6 ± 1.1 years BMI 20.7 ± 1.6 kg m^2^	• Age • Height • Weight • BMI • Single-leg squat • Lower extremity kinematics • Strength	No definition of injury provided	Self-reported by questionnaire	Significant association between limited ankle DF (OR = 1.11, 95% CI 1.02–1.20) and substantial lower extremity injuries during follow-up Significant association between limited DF ankle (OR = 1.25; 95% CI 1.03–1.52) and injury

Nineteen studies included ballet dancers [24, 26–43], two studies included contemporary dancers [44, 45], three studies included Irish dancers [46–48] and eight studies included a mixed group of dancers [49–56]. In nine studies, the dance genre was unclear [5, 57–64], and one study used dance degree students but did not state the genre [65]. With regard to the level of dance, nine studies included dancers classified as elite/professional [26–29, 37, 48, 50, 60, 62], seven studies as pre-professional [5, 33, 42, 56–59] and 21 studies as non-elite/non-professional [24, 30–32, 34–36, 38, 39, 44, 45, 49, 51–55, 61, 63–65]. Three studies used a mixed group of dancers [41, 46, 47], and in two studies, the level was unclear [40, 43].

Fourteen studies considering dancers under 18 years old [26, 34–36, 38, 39, 43, 49, 53, 55, 59, 61, 63, 64] and 16 studies including dancers above 18 years old [24, 27, 29, 40, 44–48, 50, 52, 54, 58, 60, 62, 65]. Eight studies included dancers across this age range [5, 28, 30–32, 41, 56, 57], and four studies did not report the age [33, 37, 42, 51]. Gender reporting revealed that 13 studies included females only [26, 31, 32, 34, 39, 40, 43, 44, 49, 52, 60, 63, 64], 28 studies were mixed [5, 24, 27–30, 35–38, 41, 42, 45–48, 50, 51, 53–59, 61, 62, 65] and in one study, the gender was unclear [33].

Twenty-five studies provided a definition of injury [24, 27, 28, 30–32, 38, 39, 44–48, 50–59, 62, 63], and 17 studies did not define the injury [5, 26, 29, 33–37, 40–43, 49, 60, 61, 64, 65]. In eight studies diagnosis was provided by a physical therapist/physiotherapist or doctor [26–28, 38, 44, 49, 55, 64]. In 29 studies [5, 24, 29–32, 34–36, 39, 41–43, 45–48, 50–54, 56–59, 61, 62, 65], injury was self-reported, and in three studies [33, 37, 63], the method of diagnosis was unclear. In one study, the diagnosis was provided by a ‘healthcare professional’ [60], and in one study, a nurse practitioner provided the diagnosis [40]. Five studies investigated a specific type of injury: lumbosacral pain [35], stress fractures [40], lateral ankle sprain [53], low back pain [59] and patellofemoral pain syndrome (PFPS) [63].

Fourteen studies used regression models or risk measurement [27, 31, 38, 44, 46, 47, 50, 52, 53, 56, 57, 60, 61, 65], and 18 studies used inferential analysis that did not include regression or risk measurements [5, 24, 26, 29, 30, 32, 34–36, 39, 40, 42, 43, 45, 48, 54, 58, 59]. Seven studies used both types of statistical analysis [28, 41, 49, 55, 62–64], and in three studies, the method of analysis was unclear [33, 37, 51].

### Range of Motion

Twenty-eight studies [5, 24, 26, 28–30, 32, 33, 35, 38, 39, 43, 44, 46–49, 51–56, 58–60, 63, 65] investigated the relationship between ROM and injury. With regard to genre, 11 studies included ballet dancers [24, 26, 28–30, 32, 33, 35, 38, 39, 43], one study included contemporary dancers [44], three studies included Irish dancers [46–48] and seven studies included a mixed group [49, 51–56]. In five studies, genre was unclear [5, 58–60, 63], and one study used dance degree students but did not state the genre [65]. Five studies contained dancers classified as elite/professional [26, 28, 29, 48, 60] and five studies as pre-professional [5, 33, 56, 58, 59]. Sixteen studies used non-elite/non-professional dancers [24, 30, 32, 35, 38, 39, 43, 44, 49, 51–55, 63, 65], and two studies used a mixed group of dancers [46, 47]. Nine studies used dancers under 18 years old [26, 35, 38, 39, 43, 49, 53, 59, 63], and 11 studies used dancers above 18 years old [24, 29, 44, 46–48, 52, 54, 58, 60, 65]. Six studies included dancers spanning the age ranges 9–20 years, [28], 14–18 years [5], 15–22 years [30], 14–30 years [32], 12–28 years [55] and 17–30 years [56]. Two studies did not report the age [33, 51]. Nine studies included females only [26, 32, 39, 43, 44, 49, 52, 60, 63], 18 studies were mixed [5, 24, 28–30, 35, 38, 46–48, 51, 53–56, 58, 59, 65] and one study was unclear [33].

Nineteen studies provided a definition of injury [24, 28, 30, 32, 38, 39, 44, 46–48, 51–56, 58, 59, 63], and nine studies did not define the injury [5, 26, 29, 33, 35, 43, 49, 60, 65]. Injury diagnosis was provided by a physical therapist/physiotherapist or doctor in six studies [26, 28, 38, 44, 49, 55] and was self-reported in 19 studies [5, 24, 29, 30, 32, 35, 39, 43, 46–48, 51–54, 56, 58, 59, 65]. In two studies [33, 63], it was unclear who made the diagnosis, and in one study, a ‘healthcare professional’ made the diagnosis [60].

Nine studies used regression models or risk measurements [38, 44, 46, 47, 52, 53, 56, 60, 65], and 13 studies used inferential analysis that did not include regression or risk measurements [5, 24, 26, 29, 30, 32, 35, 39, 43, 48, 54, 58, 59] to determine which factors have an association with injury. Four studies used both types of statistical analysis [28, 49, 55, 63], and analysis was unclear in two studies [33, 51].

#### Hip and Spine

‘Minor injured’ dancers had unequal hip motion (37 v 16%; *P* < 0.05) [26], and hip hyperabduction was related to foot or ankle tendinopathies and non-categorised injuries (*P* = 0.002) [49]. In dancers aged 10–11 years, hip abduction (OR 0.906; *P* = 0.021; 95% confidence intervals (CI) 0.833–0.985) was associated with PFPS. In dancers aged 15–16 years, hip internal rotation (OR 1.0603; *P* = 0.003; 95% CI 1.021–1.107) was associated with PFPS. In 10–11 year old dancers, lower back and hamstring flexibility (OR 3.542; *P* = 0.046) were a predictor of PFPS [63]. Total hip external rotation was related to injury in non-professional ballet dancers (*P* = 0.0137) [32]. Asymmetric hip internal rotation was associated with prior but not current injury with dancers demonstrating a 10° difference between the right and left limb (*P* = 0.04) [58]. At the sacrum, ballet students with an inclination angle of ≥ 30° had significantly greater (*P* < 0.05) mean low back pain intensity scores [35].

##### Meta-analysis

**Fig. 2:**
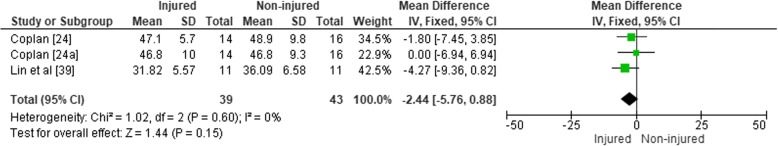
Forest plot of the comparison of hip external rotation between injured and non-injured dancers. Note: Coplan is reported twice as the author measured both right [24] and left [24a] limbs

Following a review of the included studies, only passive hip external rotation was eligible for meta-analysis [24, 39]. The pooled mean difference was − 2.44° (95% CI − 5.76–0.88; *P* = 0.15; *I*^2^ = 0%) indicating that there was no difference in this screening measurement between injured and non-injured groups (Fig. 2).

#### Knee

A study of elite female dance students reported that ‘drop outs’, some of whom suffered an injury, had a minus recurvatum (25 v 0%; *P* < 0.01) and straight legs (75 v 45%; *P* < 0.05) [26]. In pre-professional dancers, left popliteal angle was found to be correlated with injury (*r* = 0.340; *P* = 0.03) [5]. In 12–14-year-old dancers with PFPS, greater patella mobility was reported (OR 2.666; *P* = 0.029) [63].

#### Ankle

Significant differences between injured and non-injured dancers have been reported for right foot pronation with injured pre-professional ballet dancers 74% more likely to have a pronated right foot (RR 1.74; 95% CI 1.19–2.54; *P* = 0.005) and insufficient right ankle plantarflexion (RR 1.50; 95% CI 1.05–2.15; *P* = 0.037) [28]. In dance degree students, multivariate analysis of ROM during a single-leg squat identified that limited ankle dorsiflexion (OR 1.25; 95% CI 1.03–1.52) was a risk factor for substantial lower extremity injury as did univariate analysis (OR 1.11; 95% CI 1.02–1.20) [65]. Increased passive inversion range was related to ankle sprain in adolescent dancers (HR = 1.06; 95% CI 1.00–1.12) [53]. A greater percentage of hindfoot varus (OR 2.66; *P* = 0.004) and ankle dorsiflexion (OR 0.888; *P* = 0.026) existed in injured female adolescent dancers aged 12–14 years, and limited ankle plantarflexion (OR 1.060; CI 1.015–1.107; *P* = 0.009) was a predictor of PFPS in female adolescent dancers aged 15–16 years [63]. Overuse injuries occurred in female ballet dancers with decreased left ankle dorsiflexion (*P* < 0.005) [29].

#### Upper Limb

Male ballet dancers with four or more past injuries were reported to have increase elbow extension (*P* < 0.003) in comparison with other male ballet dancers [29]; however, no specific information was provided regarding injury location or limb dominance.

### Anthropometric and Posture

Twenty-seven studies investigated the relationship between anthropometric values and/or posture and injury [5, 26, 28, 29, 31, 33–40, 43, 45–50, 52, 56, 60, 61, 63–65]. Thirteen studies included ballet dancers [26, 28, 29, 31, 33–40, 43], one study included contemporary dancers [45], four studies include a mixed group of dancers [49, 50, 52, 56], three studies involved Irish dancers [46–48], five studies were unclear [5, 60, 61, 63, 64] and one study used dance degree students without further detail on the genre [65]. Seven studies contained dancers classified as elite/professional [26, 28, 29, 37, 48, 50, 60], three studies as pre-professional [5, 33, 56] and 14 studies as non-elite/non-professional [31, 34–36, 38, 39, 43, 45, 49, 52, 61, 63–65]. Two studies used a mixed group of levels [46, 47], and in one study, the level was unclear [40].

Eleven studies used dancers under 18 years old [26, 34–36, 38, 39, 43, 49, 61, 63, 64], ten studies above 18 years old [29, 40, 45–48, 50, 52, 60, 65] and four studies included dancers that spanned the age ranges 9–20 years [28], 14–18 years [5], 16–18 years [31] and 17–30 years [56] and in two studies the age was not stated [33, 37]. Eleven studies included females only [26, 31, 34, 39, 40, 43, 49, 52, 60, 63, 64], 15 studies were mixed [5, 28, 29, 35–38, 45–48, 50, 56, 61, 65] and one study was unclear [33].

Twelve studies defined the injury [28, 31, 38, 39, 45–48, 50, 52, 56, 63], but 15 studies did not define the injury [5, 26, 27, 33–37, 40, 43, 49, 60, 61, 64, 65]. Injury diagnosis was provided by a physical therapist/physiotherapist or doctor in five studies [26, 28, 38, 49, 64] and was self-reported in 17 studies [5, 29, 31, 34–36, 39, 43, 45–48, 50, 52, 56, 61, 65]. In three studies, it was unclear [33, 37, 63] who made the diagnosis. In one study, a ‘healthcare professional’ made the diagnosis [60], and in one study, a nurse practitioner provided the diagnosis [40].

Ten studies used regression models or risk measurements [31, 38, 46, 47, 50, 52, 56, 60, 61, 65], and 11 studies used inferential analysis that did not include regression or risk measurements [5, 26, 29, 34–36, 39, 40, 43, 45, 48] to determine which factors have an association with injury. Statistical analysis was unclear in two studies [33, 37]. Four studies used both types of statistical analysis [28, 49, 63, 64].

A higher incidence of spondylolisthesis was reported in ballet students who dropped out the profession in comparison with those who continued (60 v 11%; *P* < 0.05) [26]. In non-professional dancers, scoliosis was related to injury for 8 to 12-year-olds (*X*^2^ = 12.379; df = 1; *P* < 0.01), and for 13 to 16-year-olds (*X*^2^ = 30.8; df = 1; *P* < 0.01), injury risk among scoliotic dancers (8–12 years) was 1.62 greater than non-scoliotic dancers and 1.52 greater than 13 to 16-year-old non-scoliotic dancers (*P* < 0.001) [49]. In the scoliotic group, the most common injuries were to the back (47%) and knee (27%), whilst in the non-scoliotic group, it was the knee (47%) and non-categorised injuries (25.5%) (*P* < 0.001) [49]. In non-professional female dancers aged 8–16 years, there was a higher prevalence of back injuries in scoliotic dancers (OR = 19.4; 95% CI 10.2–36.4; *P* < 0.001), and significantly, more injured dancers were found among the scoliotic group (59.6%) than non-scoliotic group (37.5%) (*P* = 0.012). The RR for scoliotic dancers was higher than the non-scoliotic group for all age cohorts and significantly at the age of 9 years and from 13 to 15 years old [64]. In 15–16-year-old dancers, scoliosis was a significant predictor of PFPS (OR 5.209, 95% CI 1.353–20.052; *P* = 0.016) [63].

In a study of 806 young dancers, left thigh circumference of dancers aged 11 to 12 years was significantly larger compared to non-injured dancers (*P* < 0.05) [61]. With reference to body type, an increased likelihood of ankle injury for endomorphs was reported (OR = 1.887; 95% CI 1.433–2.312; *P* = 0.03) and increased likelihood for foot injury for ectomorphs (OR = 1.719; 95% CI 1.081–2.899; *P* = 0.05) with toe injuries more prevalent in higher body mass (OR = 1.688; 95% CI 1.410–3.121; *P* = 0.03) [31]. Twitchett et al. [60] reported a significant negative association between ‘time modifying their activity due to injury’ in elite female dancers and percentage body fat (*r* = − .614; *P* = 0.026) and (*P* = 0.039) using Spearman correlation coefficient and backward regression analysis respectively. Twitchett et al. [36] reported that in ballet students, ectomorphy was a strong predictor of the number of acute injuries sustained (*P* = 0.026), and these parameters had a significant negative correlation (*r* = − 0.37; *P* = 0.016). Significant negative correlations were observed between the dancers ‘total time off due to injury’ and percentage body fat (*r* = − 0.31; *P* = 0.048) and between the ‘total time off’ resulting from acute injury and percentage body fat (*r* = − 0.32; *PP* = 0.04) and ectomorphy (*r* = − 0.42, *P* = 0.005). The number of overuse injuries and ‘time off’ due to overuse injury was correlated with mesomorphy (*r* = − 0.38; *P* = 0.015 and *r* = − 0.33; *P* = 0.032). The mean group weight of 80% of ballet dancers with stress fractures was found to be 25% below the ideal weight (*P* < 0.005) [40]. Low back pain was more frequent in women whose body mass index was < 18.5 than those > 18.5 (*P* < 0.05) [35].

In elite adolescent ballet dancers, changes in right foot length were reported to be associated with changes in injury risk with a change of 0.5 cm associated with a moderately increased risk of injury (RR 1.41; OR 0.93–2.13) [38].

### Dance-Specific Positions

Fifteen studies investigated the relationship between dance-specific positions and injury [24, 26, 28, 30, 32, 38, 39, 41, 44, 49, 52–54, 56, 58]. Eight studies included ballet dancers [24, 26, 28, 30, 32, 38, 39, 41], one study included contemporary dancers [44], five studies were mixed [49, 52–54, 56] and one study genre was unclear [58]. Two studies contained dancers classified as elite/professional [26, 28], and ten studies as non-elite/non-professional [24, 30, 32, 38, 39, 44, 49, 52–54]. Two studies classified dancers as pre-professional [56, 58], and one study contained mixed levels of dancers [41]. Five studies used dancers under 18 years old [26, 38, 39, 49, 53], five studies above 18 years old [24, 44, 52, 54, 58] and five studies included dancers spanning the age ranges 9–20 years [28], 15–22 years [30], 14–30 years [32], 11.1–25.1 years [41] and 17–30 years [56]. Six studies included females only [26, 32, 39, 44, 49, 52], and nine studies were mixed [24, 28, 30, 38, 41, 53, 54, 56, 58].

Twelve studies defined the injury [24, 28, 30, 32, 38, 39, 44, 52–54, 56, 58], and three studies did not define the injury [26, 41, 49]. Injury diagnosis was provided by a physical therapist/physiotherapist or doctor in five studies [26, 28, 38, 44, 49] and was self-reported in ten studies [24, 30, 32, 39, 41, 52–54, 56, 58].

Six studies used regression models or risk measurement [38, 41, 44, 52, 53, 56], and eight studies used inferential analysis that did not include regression or risk measurements [24, 26, 30, 32, 39, 49, 54, 58] to determine which factors were associated with injury. Statistical analysis involved both types of analysis in one study [28].

In elite female dance students, a lack of turnout resulting in an asymmetry in the grand plié (12 v 0%; *P* < 0.005) and pronation when landing from sauté (62 v 25%; *P* < 0.05) existed in ‘minor injured’ dancers [26]. First year ‘drop outs’ had a weak sauté (12 v 0%; P < 0.05), and third and fourth year ‘drop outs’, a poor relevé (60 v 11%; *P* < 0.01) and impaired turnout of hips (pronation in plié) (40 v 0%; *P* < 0.05) [26].

A significant difference existed between injured and non-injured ballet dancers for functional turnout (*P* = 0.004) and compensated turnout (*P* = 0.006) with mean compensated turnout 25.4° in injured and 4.7° in non-injured [24]. A 1% increase in compensated turnout resulted in a 9% increase in the odds of been in the 2+ injuries group compared to the no injury or 1 injury group. Compensated turnout difference ratio was a significant predictor of been in the 2+ injuries group (OR 1.090; 95% CI 1.002–1.186; *P* = 0.046) as was muscular value ratio calculated by functional turnout/passive external hip rotation (OR 1.084; 95% CI 1.021–1.15; *P* = 0.008) [44]. Negus et al. [30] reported that the number of non-traumatic injuries was positively correlated with six of seven derived turnout variables, compensated turnout in all three positions and static dynamic turnout difference in all three positions (*r* = 0.39–0.55; *P* < 0.039). These variables were compensated turnout first position (*r* = 0.39; *P* = 0.035), compensated turnout right fifth position (*r* = 0.41; *P* = 0.028), compensated turnout left fifth position (*r* = 0.42; *P* = 0.023), static dynamic turnout difference first position (*r* = 0.39; *P* = 0.039), static dynamic turnout difference right fifth position (*r* = 0.51; *P* = 0.005) and static dynamic turnout difference left fifth position (*r* = 0.55; *P* = 0.002). Severity of non-traumatic injuries positively correlated with three of seven derived turnout variables: static-dynamic turnout difference in all positions, static dynamic turnout difference first position (*r* = 0.38; *P* = 0.043), static dynamic turnout difference right fifth position (*r* = 0.44; *P* = 0.017) and static dynamic turnout difference left fifth position (*r* = 0.47; *P* = 0.010).

Functional turnout (*P* = 0.0176) and compensated turnout (*P* = 0.0002) were related to injury in non-professional ballet dancers [32]. In university-level dancers, compensated turnout was found to be significantly related to experiencing more than one injury (traumatic and overuse combined) (*r* = 0.45; *P* = 0.04). Further analysis suggested that compensated turnout may result in more than one traumatic injury (*r* = 0.45; *P* = 0.04) whilst no significant relationship existed between compensated turnout and overuse injuries (*r* = 0.20; *P* = 0.36). Furthermore, a significant relationship existed between increased compensated turnout and low back pain (*r* = 0.50; *P* = 0.02) [54]. In adolescent ballet dancers, a 10° greater right knee alignment resulted in a moderate decrease in injury risk in the fondu (RR = 0.68; 95% CI = 0.45–1.03) and a small reduction in risk for the temps levé (RR = 0.72; 95% CI = 0.53–0.98). A 2° greater pelvic angle on the left leg for the temps levé was associated with a decrease in injury risk (RR = 0.52; 95% CI = 0.30–0.90), and a 2° greater fondu pelvic angle on the right leg was associated with an increase in injury risk (RR 1.28; 95% CI 0.91–1.80) [38]. The incorrect technique of ‘rolling in’ was related to back injuries (OR 2.166; 95% CI 1.124–4.174; *P* = 0.021) and non-categorised injuries (OR 2.707; 95% CI 1.425–5.141; *P* = 0.002) in adolescent dancers [49].

#### Meta-analysis

**Fig. 3:**
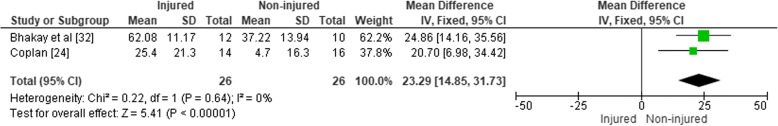
Forest plot of the comparison of compensated turnout between injured and non-injured dancers

**Fig. 4:**
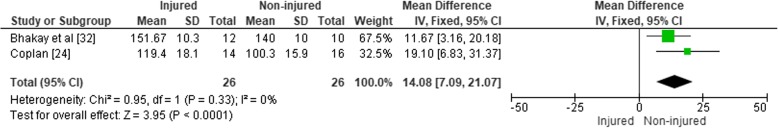
Forest plot of the comparison of functional turnout between injured and non-injured dancers

Only two dance-specific screening measurements were eligible for meta-analysis: functional turnout ROM and compensated turnout ROM [24, 32]. Another study [28] also measured functional turnout but was not included in the meta-analysis due to lack of standard deviation values, and unfortunately, attempts at requesting the data from the author were unsuccessful. The pooled mean difference of compensated turnout was 23.29° (95% CI 14.85–31.73; *P* < 0.00001; *I*^2^ = 0%) indicating injured dancers have a significantly higher compensated turnout measurement than non-injured dancers (Fig. 3). The pooled mean difference of functional turnout was 14.08° (95% CI 7.09–21.07; *P* < 0.0001; *I*^2^ = 0%) indicating that again injured dancers had significantly greater functional turnout measurements when compared to non-injured dancers (Fig. 4).

### Hypermobility

Seven studies investigated the relationship between hypermobility and injury [37, 45–48, 59, 62]. One study included contemporary dancers [45], three studies included Irish dancers [46–48], two studies were unclear [59, 62] and one study included ballet dancers [37]. Three studies classified dancers as elite/professional [37, 48, 62], and two studies reported a mixed group of levels [46, 47]. One study classified dancers as non-elite/non-professional [45] and one study as pre-professional [59]. Four studies included dancers above 18 years old [45–48]. One study included dancers below 18 years old [59], and one study used dancers spanning the age range 17–25 years [62]. One study did not report the age [37], and all seven studies used both males and females [37, 45–48, 59, 62].

Six studies defined the injury [45–48, 59, 62], and one study did not define the injury [37]. Injury diagnosis was self-reported in six studies [45–48, 59, 62] and unclear in one study [37]. Two studies used regression models or risk measurement [46, 47], and four studies used inferential analysis that did not include regression or risk measurements [45, 48, 59, 62] to determine which factors were associated with injury. The statistical analysis was unclear in one study [37].

Ruemper and Watkins [45] investigated students at a contemporary dance school and reported that 69% of students had general joint hypermobility and 33% had joint hypermobility syndrome. The total number of injuries (*r* = .331; *P* = 0.002), physical complaint injuries (*r* = .249; *P* = 0.022) and time loss injuries (*r* = .352; *P* = 0.001) were significantly correlated with the Brighton Criteria and joint hypermobility syndrome. McCormack et al. [37] investigated ballet dancers and reported that 47% of females (OR 6.75; 95% CI 1.35–33.66) and 37.5% of males (OR 7.8, 95% CI 0.90–67.37) demonstrated benign joint hypermobility syndrome as measured by the Brighton Criteria in comparison with controls. In female dancers, 78% exhibited arthralgia which was associated with skin hyperextensibility, recurrent dislocation and multiple soft tissue injuries. Only 20% of the non-benign joint hypermobility syndrome dancers exhibited arthralgia. In male dancers, 83% of those with benign joint hypermobility syndrome and 70% of the non-benign joint hypermobility syndrome dancers complained of pain.

### Clinical Diagnostic Tests

Six studies [5, 28, 39, 53, 59, 63] investigated the relationship between clinical diagnostic tests and injury and included orthopaedic testing of the foot, knee, ankle and hip [28], Thomas test [5], iliotibial band test [5, 59], anterior draw ankle [39, 53], Cumberland Ankle Instability [53], talar tilt [39] and knee joint stability [63]. Two studies included ballet dancers [28, 39], three studies were unclear [5, 59, 63] and one study included mixed genre [53]. One study [28] contained dancers classified as elite/professional, three studies contained dancers classified as non-elite/non-professional [39, 53, 63] and in two studies, the level was pre-professional [5, 59]. Five studies included dancers under 18 years old [28, 39, 53, 59, 63], and one study considered 14–18-year-olds [5]. Two studies included females only [39, 63], and four studies were mixed [5, 28, 53, 59]. Five studies defined the injury [28, 39, 53, 59, 63], and one study did not provide a definition [5]. Injury diagnosis was provided by a physical therapist/physiotherapist or doctor in one study [28], was self-reported in four studies [5, 39, 53, 59] and unclear in one study [63]. One study used regression models or risk measurement [53], and three studies used inferential analysis that did not include regression or risk measurements [5, 39, 59] to determine which factors have an association with injury. Two studies used both types of analysis [28, 63].

None of the studies reported significant findings for clinical screening tools.

### Movement Screening Tools

Six studies investigated the relationship between movement screening tools and injury [27, 46–48, 56, 57]. These tools included the Functional Movement Screen [27, 48], Functional Movement Screen and Star Excursion Balance Test [46, 47], Y Balance Test [56] and Movement Competency Screen [57]. One study included ballet dancers [26], three studies involved Irish dancers [46–48], one study was unclear on genre [57] and one study used a mixed genre of dancers [56]. Two studies contained dancers classified as elite/professional [27, 48], two studies as pre-professional [56, 57] and two studies used a mixed level [46, 47]. Four studies used dancers above 18 years old [27, 46–48], and two studies used dancers that spanned the age ranges 16–24 years [57] and 17–30 years [56]. All six studies were mixed gender and provided a definition of injury with injury diagnosis self-reported in five studies [46–48, 56, 57]. Injury diagnosis was provided by a physiotherapist in one study [27]. Four studies used regression models or risk measurement [46, 47, 56, 57], and two studies used inferential analysis [27, 48] to determine which factors had an association with injury. All other findings on movement screening tools were non-significant.

Lee et al. [57] used the Movement Competency Screen to identify injuries prospectively in full-time pre-professional dancers and reported that those dancers with a Movement Competency Score < 23 had an increased risk of injury (*P* = 0.035).

### Muscle Control, Strength, Power and Endurance

Ten studies investigated the relationships between muscle control, strength, power and endurance and injury [26, 28, 29, 33, 52, 58–60, 62, 65]. Four studies included ballet dancers [26, 28, 29, 33], and in four studies genre was unclear [58–60, 62]. One study contained a mix of genres [52], and one study used dance degree students [65]. Five studies classified dancers as elite/professional [26, 28, 29, 60, 62], two studies as non-elite/non-professional [52, 65] and three studies as pre-professional [33, 58, 59].

Three studies used dancers under 18 years old [26, 28, 59], five studies used dancers above 18 years old [29, 52, 58, 60, 65], one study [33] did not report the age and one study spanned the age range 17–25 years [62]. Six studies were mixed gender [28, 29, 58, 59, 62, 65], three studies were female only [26, 52, 60] and one study did not report the gender [33].

Five studies defined the injury [28, 52, 58, 59, 62], but five studies did not define the injury [26, 29, 33, 60, 65]. Injury diagnosis was provided by a physical therapist/physiotherapist or doctor in two studies [26, 28], self-reported in six studies [29, 52, 58, 59, 62, 65], diagnosed by a ‘healthcare professional’ in one study [60] and unclear in one study [33]. Four studies used regression models or risk measurement [52, 60, 62, 65], and four studies used inferential analysis that did not include regression or risk measurements [26, 29, 58, 59] to determine which factors have an association with injury. One study did not report statistical tests [33], and one study used both types of analysis [28].

Injured elite adolescent ballet dancers had lower extremity strength as measured by the mean score of 16 different lower limb tests than the non-injured dance group (*P* = 0.045) [28]. A significant negative correlation existed between the total number of days off due to injuries and standing vertical jump (*r* = − 0.66; *P* = 0.014) [52]. It was reported that 30% of dancers without a history of low back pain were not able to perform a correct contraction of the transversus abdominus muscle compared to 63% of dancers with a history of low back pain (*P* = 0.048), and a significant difference existed for bent knee fall out on the left leg between dancers with and without a history of low back pain (*P* = 0.049) [59]. Lumbopelvic motor control was found to predict injuries in dancers, and it was reported that the knee lift abdominal test on the right side (*P* = 0.015) and the standing bow (*P* = 0.029) were predictors of injury. A standing bow and a low pressure increase during the knee lift abdominal test were a risk for the development of lower limb injuries [62].

### Other Screening Tools

Ten studies [5, 28, 33, 46–48, 52, 56, 59, 60] investigated the relationship between other factors not appropriate for previous categories and injury. These included pain pressure threshold [46–48], number of jumps per 30 s [46–48], percentage maximum heart rate [46–48], vertical leap [46–48], Marshall Test [5], pain provocation test [59], Dance Aerobic Fitness Test [52, 60], heel balance [28], balance [48], one-leg standing test [56], unipedal dynamic balance [56] and ankle/foot risk [33]. Two studies included ballet dancers [28, 33], and three studies included Irish dancers [46–48]. In three studies, the genre was unclear [5, 59, 60], and two studies contained a mix of genre [52, 56]. Three studies contained dancers classified as elite/professional/full time [28, 48, 60], four studies used dancers classified as pre-professional [5, 33, 56, 59], one study used non-elite/non-professional dancers [52] and two studies used mixed levels of dancers [46, 47]. Five studies used dancers above 18 years old [46–48, 52, 60], and one study used dancers below 18 years old [59]. Three studies used dancers spanning the age ranges 9–20 years [28], 14–18 years [5] and 17–30 years [56], and one study did not report the age [33]. Seven studies included mixed gender [5, 28, 46–48, 56, 59], two studies used female only [52, 60] and one study did not report the gender [33].

Seven studies defined the injury [28, 46–48, 52, 56, 59], and three studies did not define the injury [5, 33, 60]. Injury diagnosis was self-reported in seven studies [5, 46–48, 52, 56, 59], not reported in one study [33], made by a ‘healthcare professional’ in one study [60] and was diagnosed by a physical therapist/physiotherapist or doctor in one study [28]. Five studies used regression models or risk measurement [46, 47, 52, 56, 60], and three studies used inferential analysis that did not include regression or risk measurements [5, 48, 59]. One study did not report the type of statistical analysis [33], and one study used both types of analysis [28].

There was a significant correlation (*r* = .590; *P* = 0.034) between the number of injuries sustained and heart rate observed at the end of the Dance Aerobic Fitness Test [60]. All other findings were reported at the non-significant level.

## Discussion

To the best of our knowledge, this is the first systematic literature review and meta-analysis to investigate which screening tools can predict injury in all genres, levels and ages of dancers.

### Methodological Quality

The mean score using the methodological quality tool was 12.2 points (range 6–17 points) with all studies being of level 4 evidence. Four studies [33, 34, 40, 51] did not provide a description of the screening tools used. As with many injury studies, the literature is limited by the varying definitions of musculoskeletal injury and by who defined the injury. Twenty-five studies [24, 27, 28, 30–32, 38, 39, 44–48, 50–59, 62, 63] provided a definition of musculoskeletal injury, but these lacked consistency in the definition. Furthermore, in only eight studies [26–28, 38, 44, 49, 55, 64], the diagnosis was made by either a physical therapist/physiotherapist or doctor. Only five studies defined the injury and had the diagnosis made by a physical therapist/physiotherapist or doctor [27, 28, 38, 44, 55]. As a minimum, it is recommended that studies should provide a definition of musculoskeletal injury and have the diagnosis made by a medical professional ideally a physical therapist/physiotherapist or doctor. The reporting of the reliability of the screening tools used is important and was reported in 13 studies [30, 31, 41–43, 47, 49, 53, 57, 62–65], but only six studies [30, 31, 43, 57, 62, 63] assessed the reliability within their own study.

### Range of Motion

A number of studies reported significant findings between ROM and injury; however, these findings were across a number of locations and comparison included different/missing musculoskeletal injury definitions, measurements taken, mix of genres, levels and ages of dancers. For those studies that demonstrated significant findings for the hip ROM, four studies used inferential analysis that did not include regression or risk measurements to identify injury [26, 32, 35, 58]. Hamilton et al. [26] reported unequal hip motion in ‘minor injured’ dancers; however, testing reliability was not reported. Hip hyperabduction was related to foot or ankle tendinopathies [49]; hip abduction, internal rotation and lower back/hamstring flexibility were associated with PFPS; hip abduction was a predictor of knee, foot and non-categorised injuries [63]. Asymmetric hip internal rotation was associated with prior injury [58], and straight leg raise ROM was increased in male ballet dancers with four or more past injuries [29]. Total hip external rotation was related to the injury in one study [32]. However, the meta-analysis [24, 39] demonstrated no significant difference in passive external hip rotation between injured and non-injured groups. The relationship between greater mean pain intensity scores and sacrum inclination angle [35] is limited by no comparative studies and the complex relationship between pain and injury.

At the knee, significant findings were limited to a correlation between left popliteal angle and injury [5]; however, injury was self-reported, and no injury definition was provided. Greater patella mobility in PFPS dancers existed compared to a control group [63]. Dancers identified as ‘drop outs’ had a minus recurvatum and ‘straight legs’ [26]; however, the reason for ‘drop out’ may not necessarily have been injury. There was a lack of consistency in the findings at the ankle with increased passive inversion [53], decreased left dorsiflexion [29], right pronation and insufficient right plantarflexion [28] all related to injury. Steinberg et al. [63] stated greater ankle dorsiflexion and hindfoot varum in dancers with PFPS, and ankle plantarflexion was a predictor of knee, back and non-categorised injuries. However, this study used age- and gender-matched dancers with and without PFPS which limits the comparison with studies of non-matched design. Limited ankle dorsiflexion during a single-leg squat was found to be a risk factor for substantial lower extremity injury [65]; however, measurement was in weight-bearing only, and no injury definition was provided. At the upper limb, significant findings were limited to increased elbow extension in male ballet dancers with four or more past injuries [29]; however, no specific information was provided regarding injury location or limb dominance. Due to the inconsistency of the results, it is unclear if ROM is a significant predictor of injury in dancers.

### Anthropometric and Posture

Limited positive findings existed between anthropometric measurements, posture and injury. A higher incidence of spondylolisthesis in ballet students who ‘dropped out’ the profession [26] may not relate to injury as for whether injury was a factor in ‘drop out’ was not reported in sufficient detail. Scoliosis was related to injury in young dancers aged 8–16 years [49] and was a predictor of PFPS in 15– 16-year-olds [63]. Scoliotic dancers aged 8 to 16 years had more total injuries, and back injuries were more prevalent [64]. In adolescent dancers, a change in right foot length was reported to be associated with increased injury risk [38]. Adolescents are subject to growth spurts and these changes may influence injury prevalence, and such age-specific findings require further investigation. The role of body type in injury was unclear in the included studies. An ankle injury may be associated with endomorphy and foot injury with ectomorphy [31]. Ectomorphy was a predictor of acute injuries whilst mesomorphy was negatively correlated with the number of overuse injuries [36]. Body fat was negatively associated with ‘time modifying activity’ [60], a term that does not necessarily equate to injury. Stress fractures were associated with below ideal weight [40]; however, this was one of the lowest scoring studies (7 points), so the methodological quality can be questioned. Left thigh circumference was larger in injured 11- to 12-year-old dancers compared to non-injured dancers of the same age [61]. One study reported a significant relationship between ‘pain’ and body mass index [35], but it is difficult to equate pain directly to the musculoskeletal injury. Due to the inconsistency of the results, it is unclear if anthropometric measures and posture are a significant predictor of injury in dancers.

### Dance-Specific Positions

The functional element of dance-specific position measurement is of potential value, and a number of significant findings were reported. Hamilton et al. [26] reported a lack of turnout demonstrated in an asymmetrical plié and pronation when landing from sauté in injured dancers; however, interpretation is limited by the use of the term ‘minor injured’ as no clear definition was provided*.* Functional and compensated turnout was greater in injured than non-injured ballet dancers [24], and an increase in compensated turnout and muscular value ratio was both predictors of increased injury [44]. Negus et al. [30] reported that the number of non-traumatic injuries was positively correlated with six derived turnout variables and non-traumatic injury severity with three derived turnout variables. Compensated turnout was related to injury in female professional ballet dancers [32] and to having more than one injury and low back pain [54]; however, both studies failed to report the reliability of screening. The incorrect technique of ‘rolling in’ and associated pronation with the patella above or medial to the first toe was related to back injuries and non-categorised injuries in adolescent dancers and may relate to trying to achieve the optimum turnout position [49]. The temps levé and fondu knee and pelvic angle were associated with injury risk in elite adolescent ballet dancers [38]. The meta-analysis provided stronger evidence that both compensated turnout and functional turnout measurements are significantly different between injured and non-injured dancers. Overall, the evidence in this review suggests that turnout measures may have the potential to be used in the identification of dancers at risk of injury.

### Hypermobility

Despite the use of recognised screening tools in the form of the Beighton Score and Brighton Criteria, limited evidence existed regarding the relationship between hypermobility and dance injury. A total number of injuries, physical complaint injuries and time loss injuries was significantly correlated with the Brighton Criteria and joint hypermobility syndrome, but no relationship existed between injury and general joint hypermobility assesses via the Beighton Score [45]. McCormack et al. [37] reported increased arthralgia in dancers with benign joint hypermobility syndrome in comparison with those without the syndrome. The methodology of this study was limited with information regarding the diagnosis and definition of injury not provided. Studies that investigate hypermobility should use the Beighton Score to define joint hypermobility and consider recent research [66] which has suggested a new spectrum of hypermobility disorders which requires investigation in dance. Due to the inconsistency of results, it is unclear if hypermobility is a significant predictor of injury in dancers.

### Clinical Diagnostic Tests

No significant findings were reported for clinical diagnostic tests, and therefore currently, it appears that clinical diagnostic tests are not a significant predictor of injury in dance.

### Movement Screening Tools

Only one study reported a significant relationship between movement screening tools and injury with the suggestion that pre-professional dancers with a Movement Competency Screen score of < 23 had an increased risk of injury [57]. However, this screening tool is relatively new, and future research with dancers is required. Analysis of studies that used the Functional Movement Screen was limited by the failure to use all the seven movements of the screen in three studies [46–48]. Interpretation of the Star Excursion Balance Test is limited to the movement in a posteromedial direction [46–48]. Therefore, it is unclear if movement screening tools are a useful predictor of dance injury.

### Muscle Control, Strength, Power and Endurance

Minimal positive findings existed within the domain of muscle strength and power with study comparison limited due to differing methods. Lower extremity strength, determined by the mean of 16 lower limb tests, was lower in injured dancers [28]; however, this study failed to consider the relative contribution of each element. Standing vertical jump as a measurement of power was negatively correlated with a total number of days off due to injuries [52]; however, injury was recorded retrospectively and via self-reporting questionnaire and therefore potentially open to recall bias. An inability to perform a correct contraction of the transversus abdominus in dancers with a history of low back pain was reported [59], and positive standing bow and a low pressure increase using a pressure biofeedback unit during right side knee abdominal lift test were found to predict injury [62]. However, limited research exists using the standing bow and knee abdominal lift test, and therefore, future research is required. No significant relationships were found between muscle endurance and injury. Due to the inconsistency of results, it is unclear if muscle control, strength and power are a significant predictor of injury in dancers.

### Other Screening Tools

Findings were limited to a significant correlation between a number of injuries sustained and heart rate observed at the end of the Dance Aerobic Fitness Test [60]. The variety of ‘other screening tools’ and limited findings suggest that it is unlikely that these screening tools predict injury. However, the positive finding for heart rate [60] requires further investigation.

### Limitations

Only three measurements were eligible for further analysis via a meta-analysis, namely passive hip external rotation ROM [24, 39], functional turnout ROM [24, 32] and compensated turnout ROM [24, 32]. Identification of which musculoskeletal screening tools may predict injury proved difficult due to the lack of standardisation of methods and reporting of data. The authors hoped to perform a meta-analysis of a number of measurements; however, this was prevented by poor reporting of methodology and variation in the measurement of parameters. Furthermore, the included literature was limited by small sample size, contrasting injury surveillance methods and risk factor identification and failure to consider confounding variables. Some studies focussed on the identification of one specific type of injury, and therefore, when contrasting these studies, care should be taken. It is also important to consider that many dancers continue to dance when injured, and although they may have pain, they may not necessarily be injured. Pain can result in dance movement modification, and this potentially could be considered in future studies. Hence, identification of which musculoskeletal screening tools may predict injury is currently difficult.

This study has provided information regarding the different genres, level of dance and ages of a dancer as all may influence the outcome of the study. Incomplete description of dancer demographics [31–33, 37, 51], inclusion/exclusion criteria [5, 24, 26–28, 31, 33, 34, 37, 38, 40, 43, 45, 49, 51, 52, 54, 55, 57, 59, 60, 62, 64] and reporting of dropouts was present in a number of studies [5, 24, 26–28, 30–33, 35–37, 39–46, 49–52, 54, 55, 60–64] which can hinder the interpretation. The lack of reported consideration of confounding variables in the study is concerning and may impact of the interpretation of results. The reliability of screening tools requires greater consideration as an unreliable tool may result in a lack of consistency in measures. Furthermore, there is a need for the average weekly dance rehearsal load to be reported to allow calculation of injury rate and exposure data as this may impact on injury rate. Information regarding performance rate, position in the company, floor surface and time point in the season are all factors that may require consideration, and studies should report the injury severity and injury duration and define the injury. The authors felt it was important to provide information on both the diagnosis and definition of injury and as such included this in the methodological scoring, as a lack of homogeneity in studies makes a comparison of incident rates and risk factors difficult. The validity and reliability of screening tools should be reported to allow determination of internal validity and inter-rater and intra-rater reliability as appropriate, and for those tools that have not had these factors determined with dancers, pilot testing is required. Studies should report their own within-study reliability. Prospective injury cohort studies are preferential in comparison with retrospective studies, and power calculations are advocated to determine sample size. Future research should consider multivariate regression models if the aim is to determine the predictors of injury and if considering multiple risk factors should control for confounding variables and consider the potential interaction of those measures that are screened. Overall, only two studies [38, 44] provided the following: (i) prospective design, (ii) an injury definition, (iii) a diagnosis by a physical therapist/physiotherapist or doctor and (iv) the use of regression models or risk measurement. These four factors could be considered good practice in investigating screening tools as a predictor of injury, and the paucity of studies that meet these requirements highlights the need for future research.

This systematic review and meta-analysis is the first to collate and critically appraise musculoskeletal screening tools as a predictor of injury across all dance levels, genres and ages. The reporting of all components of the review process allows the results to be replicated with an effective scoring tool that recognises the importance of key factors including injury reporting and reliability. Some limitations existed as the authors restricted their search to articles that were English language studies, and therefore potentially, some studies may not have been included.

## Conclusions

Evidence exists for the potential use of dance-specific positions as a predictor of injury with the meta-analysis providing evidence for the use of functional turnout and compensated turnout ROM. However, such movements are ballet specific and therefore potentially not relevant to other dance genres. Some evidence existed for measurement of hip ROM within the systematic review; however, this was not supported by the meta-analysis. The evidence for hypermobility as a screening tool is inconsistent, and there is a need to consider both the Beighton Score and the recently amended hypermobility spectrum [66]. There is a lack of studies that have utilised movement screening tools such as the Functional Movement Screen and Star Excursion Balance Test. Future studies that investigate the ability of screening tools to predict injury should be prospective, use predictive statistics, report the reliability of the tests and consider confounders. A specific definition of injury should be provided and diagnosis provided by an appropriate medical professional.

## Abbreviations

CI: Confidence interval
OR: Odds ratio
PFPS: Patellofemoral pain syndrome
ROM: Range of motion
RR: Relative risk
